# Three-Dimensional Printable Flexible Piezoelectric Composites with Energy Harvesting Features

**DOI:** 10.3390/polym15112548

**Published:** 2023-05-31

**Authors:** Mihaela Aradoaei, Romeo C. Ciobanu, Cristina Schreiner, Marius Paulet, Alina R. Caramitu, Jana Pintea, Mihaela Baibarac

**Affiliations:** 1 Department of Electrical Measurements and Materials, Gheorghe Asachi Technical University, 700050 Iasi, Romania; mihaela.aradoaei@academic.tuiasi.ro (M.A.); cristina-mihaela.schreiner@academic.tuiasi.ro (C.S.); mpaulet@tuiasi.ro (M.P.); 2 National Institute for Research and Development in Electrical Engineering ICPE-CA, 030138 Bucharest, Romania; alina.caramitu@icpe-ca.ro (A.R.C.); jana.pintea@icpe-ca.ro (J.P.); 3 National Institute of Materials Physics, Atomistilor Street 405A, P.O. Box MG-7, 077125 Bucharest, Romania; barac@infim.ro

**Keywords:** flexible composites with piezoelectric features, 3D printable flexible piezoelectric devices, energy harvesting

## Abstract

The purpose of this work was to obtain an elastic composite material from polymer powders (polyurethane and polypropylene) with the addition of BaTiO_3_ until 35% with tailored dielectric and piezoelectric features. The filament extruded from the composite material was very elastic but had good features to be used for 3D printing applications. It was technically demonstrated that the 3D thermal deposition of composite filament with 35% BaTiO_3_ was a convenient process for achieving tailored architectures to be used as devices with functionality as piezoelectric sensors. Finally, the functionality of such 3D printable flexible piezoelectric devices with energy harvesting features was demonstrated, which can be used in various biomedical devices (as wearable electronics or intelligent prosthesis), generating enough energy to make such devices completely autonomous only by exploiting body movements at variable low frequencies.

## 1. Introduction

The past 10 years have witnessed enormous interest in the efficient capture of environmental energy through the development of energy-harvesting devices that transform mechanical energy into electricity and greatly reduce our dependence on fossil fuels and CO_2_ emissions. The application segments for piezoelectric energy harvesters based on ferroelectrics have dramatically expanded during the past 5 years. For several applications, ferroelectric micro- and nano-crystals with a defined one- (1D), two- (2D), and three-dimensional (3D) shape are of great scientific and technological interest because of their spontaneous polarization as well as their shape- and size-dependent properties. The subject of moldable piezoelectric sensors with energy harvesting features is very actual; research in this area extensively reports mainly thermo-rigid devices based on epoxy, PVDF, or other composite structures, mainly with PZT powders, such as [1,2,3,4,5,6,7,8,9,10,11,12,13].

Regarding the analysis of costs vs. benefits of adapted technology and the life cycle of such devices, we note that the basic literature largely supports piezoelectric energy as being applicable in special-purpose niches with high market impact, such as wireless sensor networks (IoT). The harvesting of piezoelectric energy must be interpreted through direct indicators: the power required for the real purpose and the surplus power, which is unused and stored over time. A decision model for analyzing costs vs. benefits would include aspects such as power consumption (both active and stored), physical size/energy density requirements, storage battery technology, and working circuit technology. For example, the CC2630 Zigbee TI controller uses 1.0 µA in passive mode, whereas the CC2538 uses between 1.3 and 600 µA. In “sleep mode”, consumption is 0.1 vs. 0.4 µA.

The overall cost is also given by the relevant and precise characterization of the physical conditions that contribute to energy harvesting: the occurrence of dynamic force, vibration frequency, temperature range over time, etc. Depending on the complexity of the equipment, evaluations are made of potential improvements in energy efficiency, price, normalized price, and lifetime. As for the lifetime, the energy harvesting technologies are guaranteed for a minimum of 10 years, being made on the basis of robust materials, a sufficient element to make them very competitive on the renewable energy market, and for the technological design according to the forecasts of microelectronic applications.

From the point of view of technological feasibility, the advantages of piezoelectric generators are mentioned as being simple structures, easy to manufacture on a large scale (mass production), on the one hand, and easy to implement in electronic systems of the ‘energy harvesting’ type.

The new trend is defined by flexible energy-harvesting devices, which are yet more difficult to manufacture. Some achievements are presented as [14,15,16,17,18,19,20,21,22,23,24,25,26,27,28], but they are practically never combined with 3D printing technology, which is the interest of our research and the subject of the actual paper. In general, a wide range of composites made of ceramic powders and polymers can be processed by 3D printing technology; however, the receipt at the nanoscale is a key factor in successful part fabrication. The main unmet need is related to the difficulty of adapting any new variant of composite with individual thermo-mechanical characteristics on any 3D printing machine, and there is a must—achieved only by an extended R&D activity—the technological balance between the desired features of filament material, on the one side, and 3D printing capabilities, on the other side, in order to create the best characteristics of the obtained component. The values of material density, viscosity, and surface tension must be correlated. When the ratio is too small, viscous forces predominate, which implies high pressure for ejection; inversely, if this ratio is too large, a continuous column is ejected, which can lead to the formation of satellite drops behind the main drop. The rheology of thermoplastic nano-composite and its behavior at different processing temperatures are problems related to compound melting temperature, stability in the quasi-liquid phase, and final product isotropy. If mixed dissimilar materials are used for nano-composites, e.g., ceramic powder/CNT with a polymer matrix, even if the product is homogenous and stable after extruding—when the filament is generated—it may suffer important physical modifications during the subsequent stage of 3D printing technology. The flowability of powders in quasi-liquid phases is an essential parameter for 3D processing. Sufficient flowability allows the building of high 3D resolution [29,30,31].

The 3D architectural design of printable piezoelectric components involved a good knowledge of the adsorption of thermoplastic polymers on the surface of ceramic nanoparticles. In the stages prior to the realization of the 3D printable flexible piezoelectric composites with energy harvesting features, the following results were reported by the authors [32,33,34] as preliminary methods of preparing films containing thermoplastic polymers and their composites with BaTiO_3_, along with some optical and structural properties.

Our study presents an example of good practice under the circumstances that it is obvious that by an extruding process pellets from thermoplastic composites may be manufactured, even if the inorganic powder content goes towards 80–90%, but it is also well known that the manufacture of rigorous filament in terms of diameter tolerance and reasonable length from thermoplastic composites with more than 10–15% inorganic powders is extremely difficult, mainly due to the fragility of the filament, which in many cases breaks itself under the reeling operation. That is why a tailored compounding of thermoplastic olefin with thermoplastic polyurethane was used. On the other hand, at higher powder contents, the filament homogeneity can suffer important changes under cooling operations due to dilatation, exfoliation, or delamination processes. Another major difficulty noticed by many practitioners refers to the thermal printer adaptation for such composites with a high content of inorganic powders, which makes the actual commercial printers useless for such applications, mainly when higher printing precision (micrometer scale) is compulsory.

The innovation presented by the present study includes a clear demonstration of the conditions imposed on such composites to become 3D printable, i.e., starting from the compounding and filamentation stage, through the 3D thermal deposition of composite filament with no clogging of the nozzle, no problems with multilayer deposition, and no 3D printing defects, to generating structures of rectangular network type with micro-meter precision, along with innovative tests to demonstrate their use as piezoelectric sensors within a dedicated signal processing circuit and the use of virtual instruments to assess their energy harvesting features.

## 2. Materials and Preparation Methods

### 2.1. Materials

In order to obtain the composite structures, the following raw materials were used: a thermoplastic olefin TPO (for the presented study, polypropylene TIPPLEN H 318), a thermoplastic polyurethane TPU (Estane 58,887 TPU), and BaTiO_3_ powder of a maximum 2 micron dimension from Sigma Aldrich (Merck KGaA, Darmstadt, Germany). The present study completes and particularizes the previous research presented in [34] and refers only to TPO:TPU 2:1 type composites with BaTiO_3_ powder content up to 35% in order to emphasize their piezoelectric features.

### 2.2. Processing Equipment

The polymers as powders (TPU and TPO—herein polypropylene) and the BaTiO_3_ powder were homogenized by mixing for one hour in a cylindrical mixer with a 1.3 L capacity TURBULA T2F type with a rubber ring holding device, and the rotation speed was 40 rpm. In this way, we sought to obtain a uniform distribution of the components of the mixtures throughout the structure without using specific additives or adhesives for compatibility. Processing conditions involved a rotation speed (in counter-rotation) of the extruder of 95 rpm and a feed speed from the feed hopper of 450 rpm.

**Figure 1:**
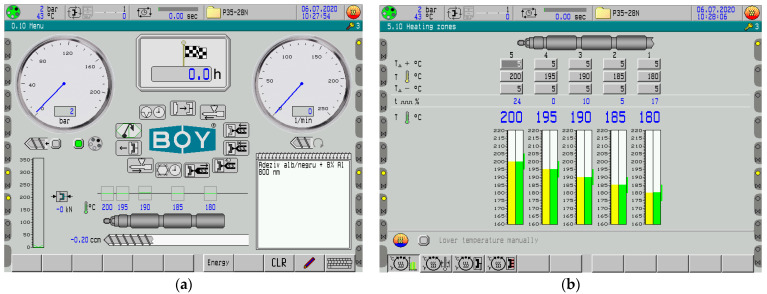
(**a**) The interface of the injection machine, and (**b**) the temperature regime on the areas of the cylinder of the injection machine.

The injection of composite was performed on a Dr. Boy 35A injection machine (Dr. Boy GmbH & Co. KG, Neustadt-Fernthal Germany) with the following characteristics: a screw diameter of 28 mm, an L/D ratio of 18.6 mm, a calculated injection capacity of 58.5 cm^3^, a maximum material pressure of 2200 bar, and a real injection capacity minimum of 500 mm. The interface of the injection machine for obtaining the composite materials and the temperature regime on the areas of the cylinders of the injection machine are briefly presented in Figure 1.

### 2.3. Characterization Equipment

The characterization of the composite materials was carried out with the use of the following equipment:

- The photoluminescence (PL) spectra of composites were recorded with a Fluorolog-3 spectrophotometer, FL3-2.2.1 model, from Horiba Jobin Yvon (Palaiseau, France), with some expertise related in [35].
- SEM optical scanning microscopy was performed with a field emission and focused ion beam scanning electron microscope (SEM) model Tescan Lyra III XMU (Brno—Kohoutovice, Czech Republic).
- The AFM analysis was performed with a WIKO NT 1100 type interferometric microscope in accordance with ISO 10109-7:2001 [36].
- Dielectric features were determined by using the Broadband Dielectric Spectrometer (Novocontrol GMBH), which encompasses an Alpha frequency response analyzer and Quattro temperature controller with tailored measurement cells. The manufactured samples were sandwiched between two copper electrodes of 20 mm diameter and placed inside the temperature-controlled cell [37].
- The piezoelectric features were measured using an Aixact TF Analyzer 2000-Electric Hysteresis Curve Lift System (static and dynamic hysteresis); the voltage that can be applied to the sample is +/− 100 V to +/− 10 kV). The device measures bulk samples (maximum diameter of 20 mm and a maximum thickness of 1.8 mm). The hysteresis curves were raised starting with a frequency of 0.1 Hz at an electric voltage 20% lower than the breakdown voltage of the samples. Simultaneously, the displacement of the sample can be measured with a laser interferometry system.
- TG/DSC thermal analysis, performed on a STA 449 F3 Jupiter TG-DSC simultaneous thermal analyzer, Netzsch (Selb, Germany), working in the temperature range up to 1550 °C, in an inert, oxidizing, reducing, static, or dynamic working atmosphere. The device is provided with a vacuum system with a maximum of 10^−2^ mbar.

## 3. Results and Discussion at the Composite Level

### 3.1. Photoluminescence Spectrum

The photoluminescence spectrum of thermoplastic composites exhibits a maximum at 462 nm when the excitation wavelength used to record the photoluminescence spectrum is 350 nm. Anisotropy (r) and bond angle (ϕ) of macromolecular compounds, adsorbed on the surface of inorganic particles, can be calculated using anisotropic photoluminescence with the following formulas:

r = (I_vv_ − GI_vH_)/(Ivv + 2GI_vH_) r = 0.4[(3cos^2^ϕ − 1)/2],

where I_vH_ corresponds to the photoluminescence intensity when the polarizer is positioned vertically for excitation and horizontally for emission in the spectrophotometer; G = I_HV_/I_HH_, where I_HV_ corresponds to the light intensity measured when the polarizer is positioned horizontally for excitation and the polarizer is positioned vertically for emission.

**Figure 2:**
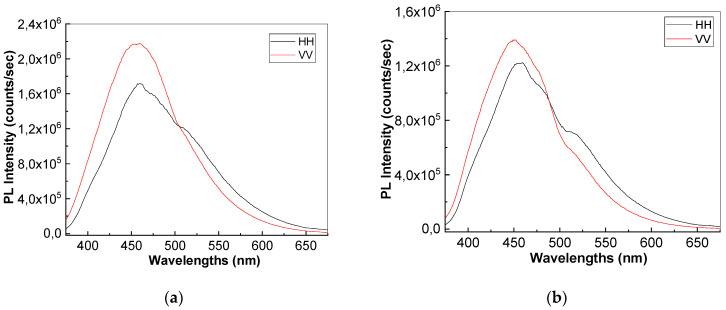

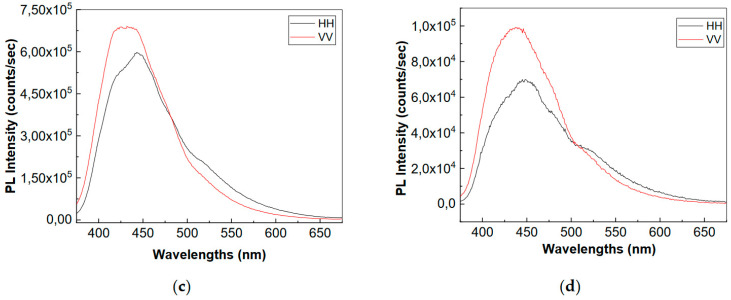
Photoluminescence spectra in polarized light of the following: (**a**) TPU:TPO 2:1; (**b**) TPU:TPO 2:1 + 12% BaTiO_3_, (**c**) TPU:TPO 2:1 + 25% BaTiO_3_, and (**d**) TPU:TPO 2:1 + 35% BaTiO_3_.

The photoluminescence spectra in polarized light of composites with BaTiO_3_ nanoparticles having concentrations of 12 wt.%, 25 wt.%, and 35 wt.%, respectively, are presented in Figure 2. In Table 1, the values of the intensity of the photoluminescence spectra (PL) are presented, taking into account the way of mounting the polarizers for excitation and emission. According to Table 1, the gradual decrease in the intensity of the PL spectra of TPU:TPO, regardless of the way of mounting the polarizers, as the concentration of inorganic nanoparticles increases, indicates the role of BaTiO_3_ as quenching agent of polymeric matrix’s PL. This result is in agreement with our previous paper [34]. Considering the I_VV_, I_VH_, I_HV_, and I_VV_ values experimentally obtained, which are presented in Table 1, the r and ϕ values in the case of: (i) TPU:TPO 2:1 are equal to 0.302 and 23.70; (ii) TPU:TPO 2:1 + 12% BaTiO_3_ are equal to 0.0862 and 46.30; (iii) TPU:TPO 2:1 + 25% BaTiO_3_ equal to 0.078 and 47.10; and (iv) TPU:TPO 2:1 + 35% BaTiO_3_ equal to 0.029 and 51.80, respectively. In regard to the anisotropy, the composite with 12% BaTiO_3_ presents the maximum value and is explained by a more homogenous dispersion of particles with different spatial orientations, which is a phenomenon expected for homogenous dispersions with low quantities of particles. In regard to the bond angle, the maximum value is reached by the composite with 35% BaTiO_3_, which is explained by the composite architecture and will be further analyzed by SEM optical scanning microscopy. The value of r is less than 0.4, a fact that indicates that the excitation and emission transition dipoles are not aligned, which explains the complex dielectric polarization presented further in the paper. This aspect originates in the exchange reaction of the repeating units of the TPU-type polymer with BaTiO_3_ [33] because it is expected for the dipole of TPU to make the main contribution to both interfacial and dipolar polarizations.

### 3.2. SEM Structural Analyses

**Figure 3:**
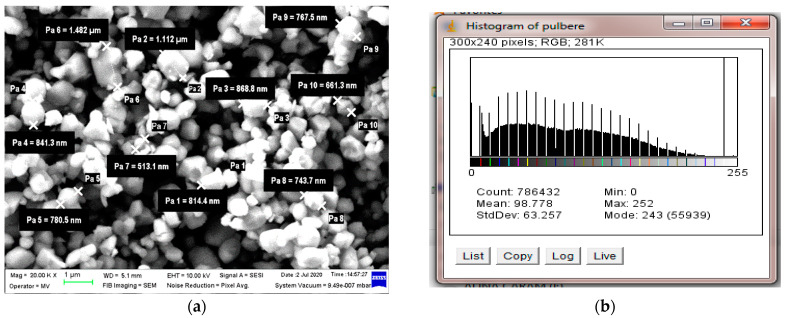

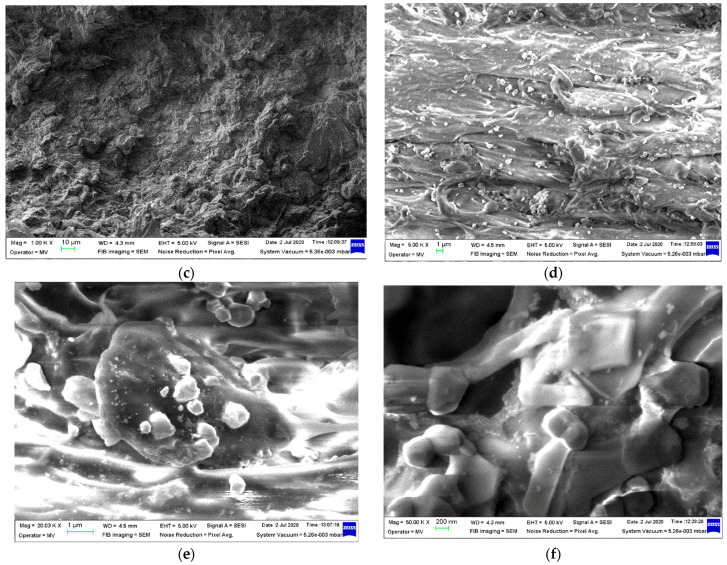
Micrograph of BaTiO_3_ with 50,000× magnification (**a**), and related BaTiO_3_ particle histogram (**b**); Micrographs of composite with 35% BaTiO_3_ with magnifications (**c**) 1000×, (**d**) 5000×, (**e**) 20,000× and (**f**) 50,000×.

SEM structural analyses were performed to highlight the degree of homogeneity of the obtained materials. These analyses were performed on the field emission source and focused ion beam scanning electron microscope. The average dimension of BaTiO_3_ particles was about 1 μm, Figure 3a,b.

It can be generally seen that the filler is well homogenized within the polymer structure with a uniform dispersion, Figure 3c–f. Though slight agglomerations of the filler particles may appear, which may induce a slight inhomogeneity; however, in small, negligible areas, this aspect can be remediated by a longer mixing time and homogenizing the BaTiO_3_ powder at a higher powder content.

### 3.3. Atomic Force Microscopy (AFM) Analysis

**Figure 4:**
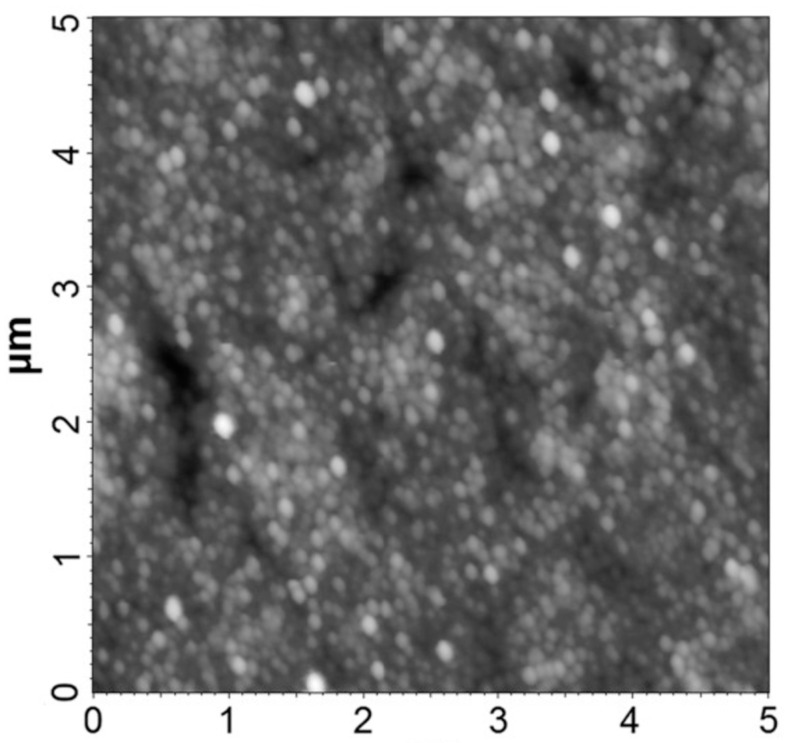
AFM microscopy image of the composite with 30% BaTiO_3_.

Basically, the experimental models made of polymer composite materials are very difficult to withstand atomic force microscopy tests because, for the AFM microscopy tests, a surface with nanometric roughness (preferably approx. 50 nm) is required. The roughness was determined as the average value between three measurements performed on the same sample in its central area at a focal distance of 90 μm. Roughness was obtained in the range of 75–450 nm, which gives some minor errors under the circumstances presented as above. In Figure 4, an AFM microscopy image is presented for the composite with 35% BaTiO_3_, which is considered more relevant due to the higher content of powder, and the resulted roughness was below 100 nm. The roughness of such composites may be relevant in the case of producing filaments for 3D printing purposes, and the obtained value is in line with the requirements for such applications.

A quasi-uniform distribution of the particles on the surface of the composite material can be observed as a whole, eventually depending on the size of the particle and its concentration. In the case of inorganic powders (BaTiO_3_), the effect appears more blurred, but there is also a subjective cause given by the optical contract of such powders. These preliminary conclusions from the AFM analysis can be clearly correlated with those from the SEM analysis.

### 3.4. Dielectric Tests

**Figure 5:**
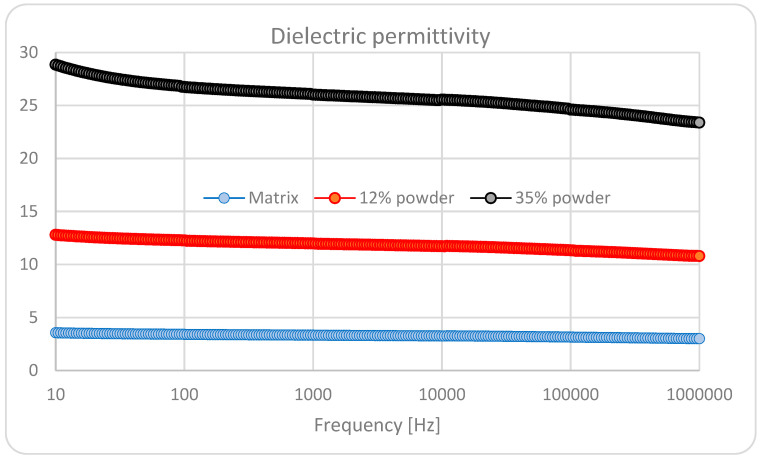
Relative permittivity.

**Figure 6:**
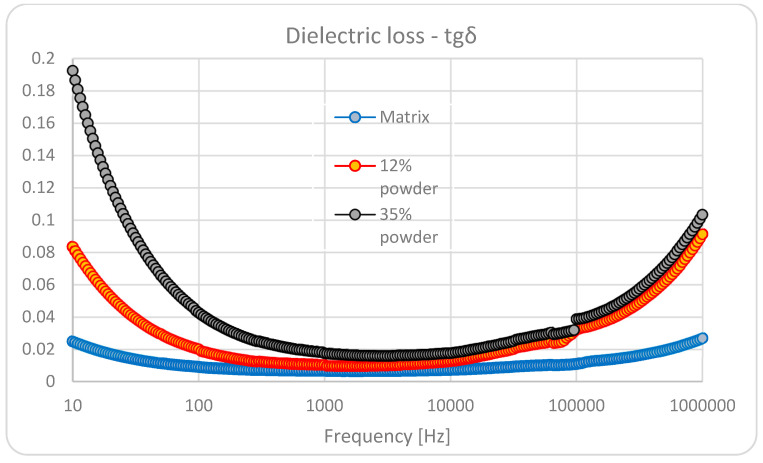
Dielectric loss (Tg Delta).

The dielectric characteristics of the composite materials are presented in Figure 5 and Figure 6, for the blended matrix the thermoplastic composite with 12% BaTiO_3_, and the thermoplastic composite with 35% BaTiO_3_.

It is obvious that the addition of BaTiO_3_ dramatically increases the dielectric permittivity, from about 3.5 of the matrix towards about 13 for an addition of 12% BaTiO_3_ and finally towards about 28 for an addition of 35% BaTiO_3_ at 10 Hz frequency. With the increase in frequency, all permittivity values are decreasing, with the largest decrease being for the composite with the higher content of BaTiO_3_.

In regard to the dielectric loss, at lower frequencies, the effect of interfacial polarization is detrimental, with a very significant value for an addition of 35% BaTiO_3_. Herewith, we speak about three types of complex interfaces: one related to blended matrix and the others related to polymers and BaTiO_3_. That is why we can notice an increased value at low frequencies for blended matrixes as well. At medium frequencies in the kHz domain, the difference is not so relevant, but at higher frequencies, over 50 kHz, the dielectric polarization effect is obvious, with relevant higher values for the highest content of BaTiO_3_.

On the other hand, when analyzing the dielectric loss characteristics of the samples with BaTiO_3_, an interesting variation effect with frequency may be noticed in the vicinity of the 100 kHz frequency domain. Here we can speak about a saltation of the tgδ characteristic, correlated with the architecture of composites, which induces an additional ionic-dipolar conjugated polarization, e.g., a displacement due to the balance between the resonance and anti-resonance frequencies. Such phenomena explain the use of such composites with piezoelectric powders as resonators/filters for tailored applications in the radiofrequency electronic field. In our case, even if such phenomena occur, the low quantity of BaTiO_3_ powder cannot make the respective composites relevant candidates for such electronic applications. But this phenomenon can be further exploited in relation to 3D printing in order to achieve a quasi-4D effect with frequency. Such an effect may become useful when collecting electrical signals from 3D printable flexible piezoelectric devices to be obtained from such composites for energy harvesting purposes, mainly when they are used for biomedical applications—communications at MHz frequencies.

As long as for reasonable piezoelectric effects the concordance of the highest value of three parameters is needed, i.e., the content of BaTiO_3_, dielectric permittivity, and dielectric loss at lower frequencies, we may admit that the most relevant sample is the one with 35% BaTiO_3_, which will be analyzed with priority as follows.

### 3.5. Piezoelectric Characteristics

**Figure 7:**
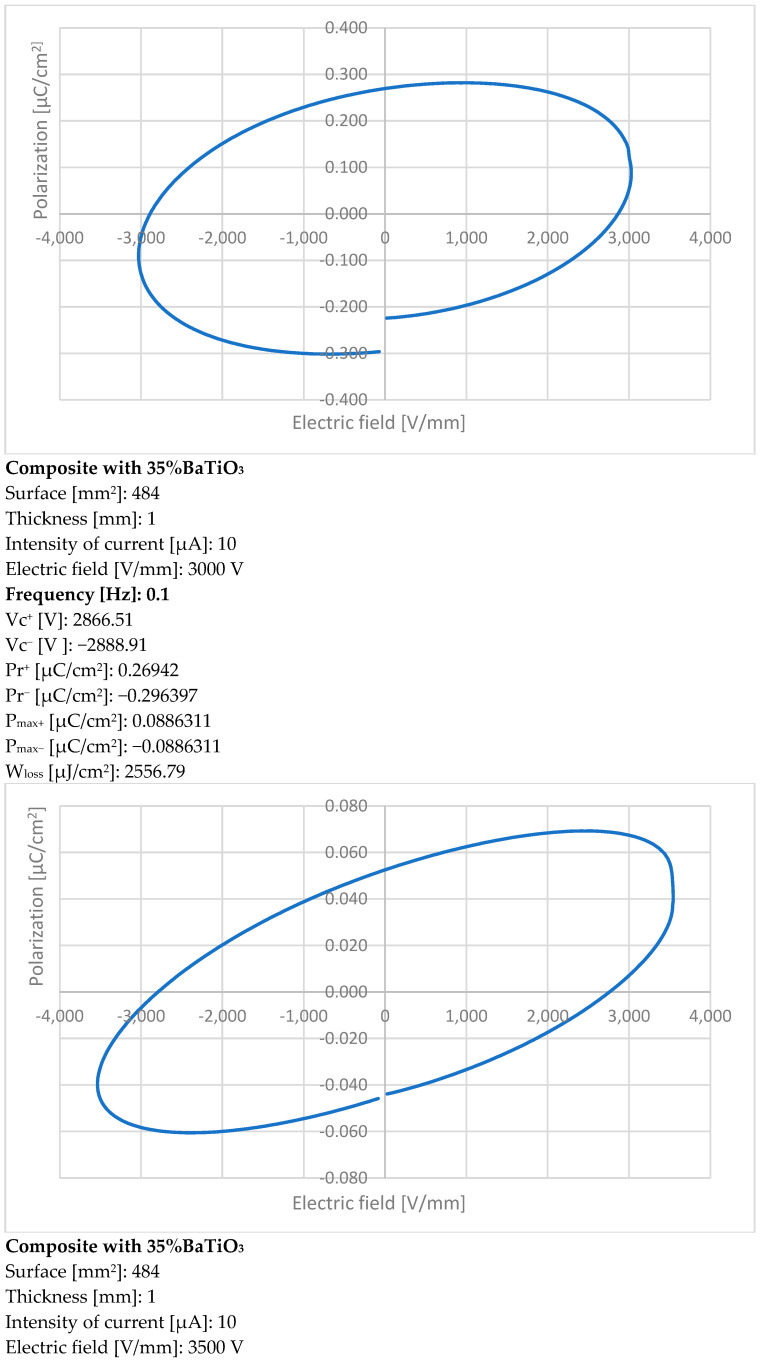

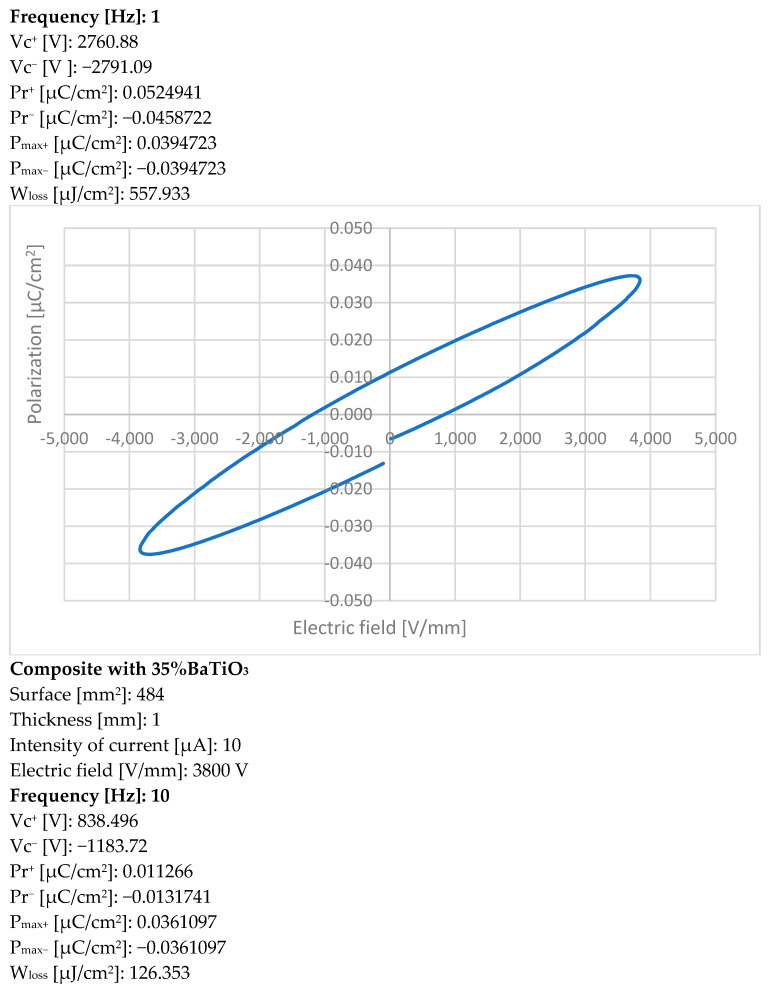
Polarization characteristics at 0.1, 1, and 10 Hz.

The piezoelectric characteristics for the composite with 35% BaTiO_3_ without preliminary activation under a tailored electric field are presented in Figure 7 at three different frequencies. The respective frequencies were chosen taking into account the potential use of such composites for 3D printable flexible piezoelectric devices with energy harvesting features for biomedical applications, i.e., in conjunction with wearable electronics or intelligent prosthesis.

The values in each table are specific for the hysteresis curve obtained at different frequencies and clearly indicate an alternating current ferroelectricity of the composite.

The values expressed as positive P_max_ and negative P_max_ show the symmetry of the curve at the maximum polarization level. The values expressed as negative Pr and positive Pr show a small difference due to the fact that the hysteresis curves do not fully close, but this aspect is characteristic of BaTiO_3_. Vc values depend on applied voltage and can be further correlated with the size of the granules, but this is not relevant in our case. Depending on the voltage applied to the samples, different hysteresis curves are obtained, and the maximum polarization is correlated with the applied voltage values. By increasing the frequency value of the applied voltage, a flatter curve is obtained. The angle of inclination of the curve depends on the concentration of the inorganic powder; e.g., an angle of about 45° is observed at 10 Hz for the addition of 35% BaTiO_3_.

The hysteresis losses, the most relevant parameters for piezoelectric analysis, are correlated with the volume of the hysteresis curve and expressed by W_loss_. The ideal curve for analyzing the piezoelectric features of the composite is the one at 0.1 Hz, where the W_loss_ value reaches 2556.79 μJ/cm^2^.

The results are remarkable for a material without preliminary activation under a tailored electric field, and the relatively high polarization at low frequencies is in line with body reactions, which can be used as a source of energy harvesting via the piezoelectric effect. Further, the features of such composites with activation under a tailored electric field will be presented after the related piezoelectric devices are obtained by 3D printing technology from the filaments achieved with composites with BaTiO_3_.

### 3.6. Thermal Stability

**Figure 8:**
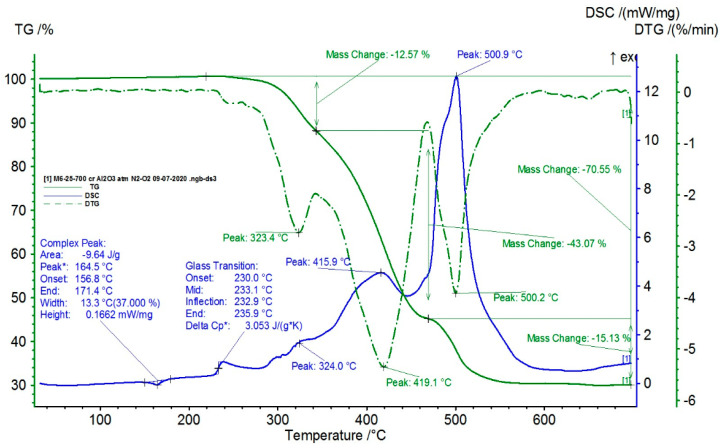
TG and DSC variation curves for the sample with 35% BaTiO_3_.

TG-DSC (Differential Scanning Calorimetry) independently measures the heat flow rates between a sample and a reference subjected to the same temperature program (isothermal or dynamic). The difference in heat flow between the sample and the reference, which are heated (or cooled) over a certain temperature range, is then determined, and this difference is plotted as a function of temperature. The direction of the thermal flow towards the DSC sensor is well defined and reproducible [38]. The results for the composite with 35% BaTiO_3_ are presented in Figure 8.

For such composite material, in addition to glass transition processes and chemical oxidation processes, there is also a first-order phase transformation process (melting) due to the TPO (PP) melting process. The maximum melting temperature is around 170 °C.

## 4. Development of 3D Printed Flexible Piezoelectric Structures

**Figure 9:**
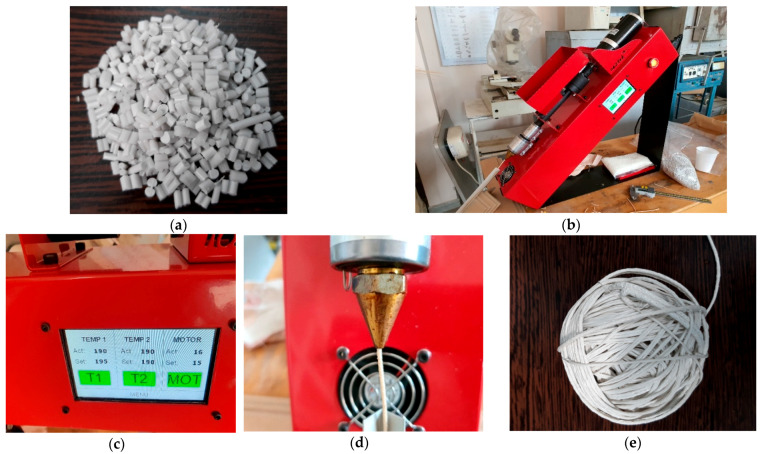
Specific filaments for 3D printing with a diameter of 1.75 mm from the composite pellets with 35% BaTiO_3_; (**a**) pellets; (**b**) laboratory extruder; (**c**) temperature control; (**d**) filament generation through the extruder head; (**e**) filament reel.

The development of flexible and elastic piezoelectric energy capture devices based on customized composite elastomers and 3D printing technology involved modeling by fused deposition of composite materials in the form of filaments. The preliminary tests for making specific filaments for 3D printing with a diameter of 1.75 mm were carried out on a small laboratory extruder (Figure 9) from extruded pellets. It is obvious that by an extruding process pellets from thermoplastic composites may be manufactured, even if the inorganic powder content goes towards 80–90%, but it is also well known that the manufacture of rigorous filament in terms of dimeter tolerance and reasonable length from thermoplastic composites with more than 10–15% inorganic powders is extremely difficult, mainly due to the fragility of the filament, which in many cases breaks itself under the reeling operation. On the other hand, at higher powder contents, the filament homogeneity can suffer important changes under cooling operations due to dilatation, exfoliation, or delamination processes. The purpose of the experiment was to develop the optimal extrusion technology, i.e., to account for the optimal temperatures and the speed of pulling the filament to make filaments with rigorously constant dimensions at higher quantities of inorganic powders.

In this context, adequate thermal control of the extruder was important, with the used temperature being between 180 and 190 °C and more composite recipes being tested [34]. Finally, the filament from the composite material with 35% BaTiO_3_ resulting from the extrusion operation was elastic, mainly due to the innovative addition of TPO, even close to the elasticity of rubber, but it provided good features to be used for 3D printing applications as well and presented a reasonable variation in a maximum diameter of 10%. The optimal temperatures were around 190 °C with a pulling speed of 15 cm/min. The filament was homogenous, with a smooth surface and no mechanical defects, and had reasonable winding features (Figure 9).

**Figure 10:**
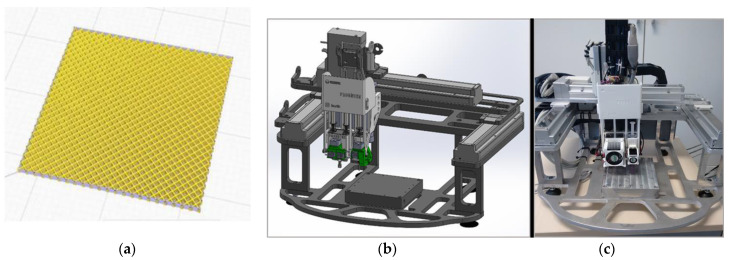
Three-dimensional printed experimental structural model and the dedicated printer; (**a**) rectangular parallelepiped mesh; (**b**,**c**) images of the tailored printer (face and side view).

The preliminary 3D printing tests for the realization of structural models were performed using an adapted laboratory 3D thermal printer with a maximum working surface of 120 × 120 mm^2^. Other parameters taken into account for printing the models were the viscosity and density of the topical filament. Preliminary testing of the 3D printing parameters is listed in Table 2. A comparison was made between the experimental filaments and the commercial ScotchBlue™ Original Painter’s Tape-type PLA filaments and revealed similarities and compatibility by testing the successive depositions of the two filaments; hence, the base deposition support can be made with the commercial one (Figure 10).

Using the Solid Works CAD program or other specialized CAD software, 3D models can be designed, e.g., one of the rectangular parallelepiped network types, which are in fact a 25 × 25 × 1 mm mesh type. After the creation of the CAD model, it is transferred to the laboratory 3D thermal printer software. The experimental structural model is presented in Figure 10.

**Figure 11:**
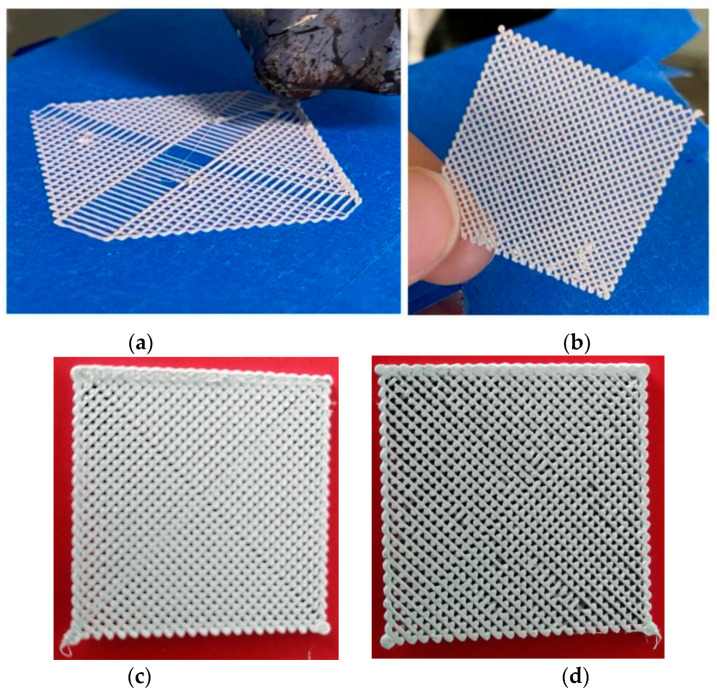
Three-dimensional printed devices for energy harvesting as precision mesh structures; (**a**,**b**) tests with progressive deposition of filament with different angles; (**c**,**d**) final mesh structures with different densities.

It was shown that the deposition of composite filament with 35% BaTiO_3_ was a convenient process with no clogging of the nozzle, no problems with multilayer deposition, and no 3D printing defects. In the end, very good precision structures were obtained experimentally by adjusting the deposition parameters. Even precision mesh structures were made at an angle of 90 compared to the initially planned one of 45 and at deposition densities higher than 50% (Figure 11), to be further tested as basic devices for energy harvesting. As a final comment on this chapter, we can estimate that the mesh structures manufactured by thermal printing provide similar features as homologue structures described in the literature, e.g., in [39], but these are achieved by less productive and more complicated and expensive processes, i.e., stereolithography. Therefore, the advantages of the composite recipe and technology for 3D printing described in this paper are obvious.

## 5. Testing the Energy Harvesting Features of 3D Printed Devices

**Figure 12:**
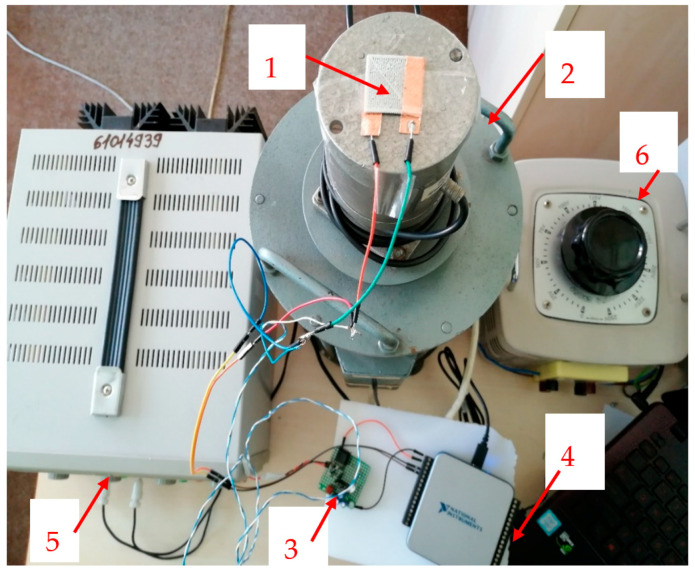
The test stand used for the production and acquisition of the signal includes the following: 1—device/sensor; 2—vibration creation equipment; 3—capacitor charging circuit; 4—NI USB acquisition board—6001; 5—direct current source (constant voltage 24 V); 6—autotransformer (alternating voltage supply)—for vibration increase/decrease regime.

For the development and integration of the signal processing circuits and the demonstration of the functionality of the energy harvesting features of 3D printed devices, a special test stand was developed, as in Figure 12, to produce tailored vibrations to activate the devices—here with functionality as piezoelectric sensors. To realize sensorial features, the mesh structures are covered on both sides with self-adhesive copper strips in order to collect the electrical charges obtained by piezoelectric phenomena.

To generate the vibrations, specialized equipment (2) with two windings was used (composed of a fixed part and a mobile part, the mobile part being connected to the fixed one by means of an elastic mechanical suspension), supplied with a constant voltage of 24 V dc from the direct voltage source (5) and also with alternating voltage from the autotransformer (6). The vibration force is proportional to the currents absorbed by the two windings, according to Equation (1):

F_din_ = kI_cc_I_ca_ (1)

The vibration frequency was up to 50 Hz, and their amplitude was proportional to F_din_. When the position of the cursor of the autotransformer is changed, the increase in the alternating voltage applied to the device (2) is achieved, and the mobile part (on which the sensor (1) is placed) will start to vibrate, and thus a signal will be generated at the two terminals of the self-adhesive copper strips of the sensor. Further, this signal is used to charge a capacitor with the role of an energy harvester, and if a resistor R is connected to the output of the sensor, the effective value U of the voltage of the oscillations generated by the sensor can be measured, with the power value to be determined with Equation (2):

P = U^2^/R (2)

**Figure 13:**
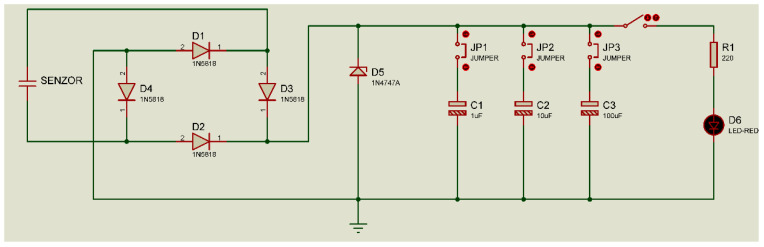
The circuit for acquiring and rectifying the voltage from the sensor.

In our case, a very common electronic processing module, as presented in Figure 13, was used to collect the energy of the oscillations generated by the sensor and identified in Figure 12 as 3—capacitor charging circuit.

The output from the sensor is applied to a rectifier bridge made with 4 Shottky diodes of type 1N5818 in order to achieve a continuous charge process only and avoid the reverse bias effect.

The time variation of the voltage *u*(*t*) on the capacitor is given by Equation (3), where *U* is the voltage applied to the capacitor resulting from the rectification and *R* is the equivalent load resistance of the capacitor.

(3) $$u\left(t\right)=U\left(1-e^{-\frac{t}{RC_{H}}}\right)$$

The maximum energy *E* accumulated in the capacitor to which the voltage *U* is applied is given by Equation (4).

(4) $$E=\frac{C_{H}U^{2}}{2}$$

**Figure 14:**
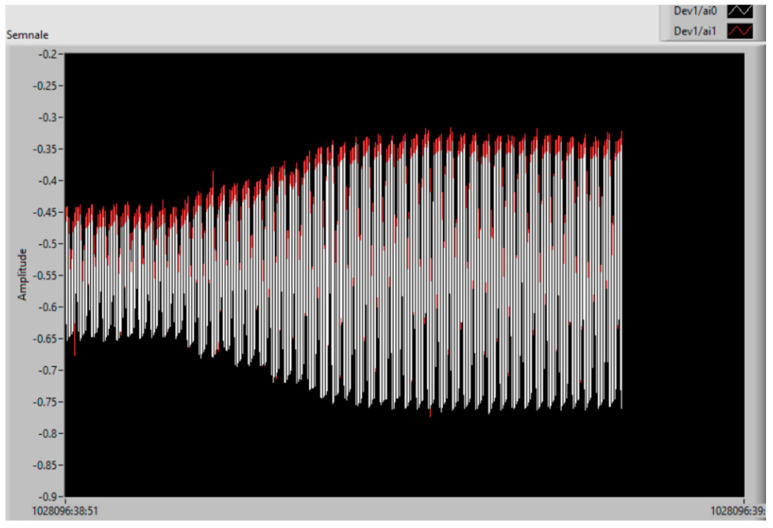
The analog signals by increasing the voltage/vibration level: sensor (red); charging capacitor (white).

The analog signals when increasing the voltage of the vibration device, i.e., the vibration amplitude level increases, are presented in Figure 14. The signal on the sensor is represented by red oscillations, and the signal on the charging capacitor by white oscillations. The scale is related to signal period, and so the interpretation of the results is difficult without analyzing the real charging phenomenon.

**Figure 15:**
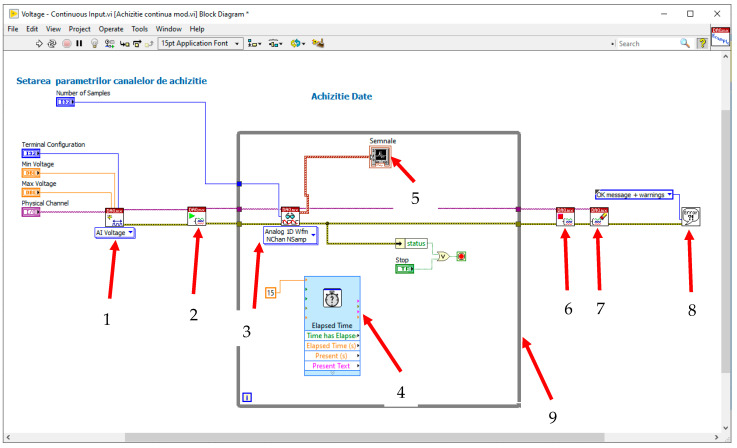
Block diagram of the instrument.

That is why intelligent signal processing was embedded by using a virtual instrument (VI) developed under the LabVIEW graphical programming environment [40], briefly presented in Figure 15. After the signal has been acquired with the acquisition board, it is digitized and then transferred to the PC via the USB port. From the USB port, the acquired signal is then processed and displayed using the VI. Block 1 is the data acquisition board setup in subVI. The measured quantity will be set; in our case, it will be “AI Voltage”, i.e., the analog voltage. Block 2 is a block used to open a signal acquisition “task”. If this subVI is not used, it is possible for the application to start an unsynchronized signal acquisition by itself. If the “DAQmx Start Task VI” subVI and the “DAQmx Stop Task VI” subVI are not used when the “DAQmx Read VI” subVI or the “DAQmx Write VI” subVI are used multiple times (how it would be in a loop) and the acquisition starts and stops repeatedly, starting and stopping a purchase repeatedly reduces app performance. For a continuous acquisition of the signal, the acquisition block (3) is inserted into a “WHILE” loop (9) that will run until an event occurs that will interrupt the running of this loop, either an error or pressing the “STOP” button.

Block 3 is the actual signal acquisition block and has been configured for the acquisition of analog signals. Because we are acquiring signals on several channels, “Multiple Channels” will be selected. Block (4) is a timer used to synchronize the acquisition with the display. After acquiring the signals, these vectors in which the values are stored are displayed value by value on display 5, after which these values are interpolated. These values will be presented on the *Y*-axis vs. the *X*-axis, which will show the effective time.

SubVI (6) is used to close the “task” and is opened by block (2). Blocks (2) and (6) will always be used together. The SubVI (7) will clear all values stored in the 1D-type variables to prepare the VI for the next acquisition. The SubVI (8) will display an error message if errors occur. A sample count of 100 has been set, so we will store the values in a 1D vector variable.

The front panel of the signal acquisition instrument was designed to simulate different functional regimes of the energy harvester, i.e., the resistor value, the time, the range (limits) of the input voltage, and the number of signal samples to be processed vs. time.

Two signals are acquired, one directly from the sensor on channel A1 (the waveform is red) and the signal from the capacitors on channel A0 (the waveform is white), the latter being displayed separately for an analysis vs. charging time and charging regime.

The first experiments are performed with the sensor without a preliminary activation with an electric field.

**Figure 16:**
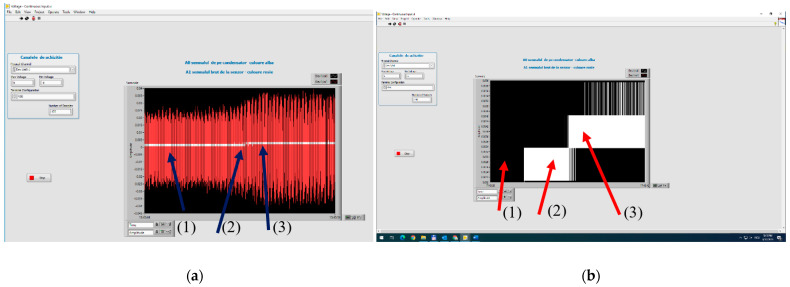
Increasing vibration level at the test equipment (**a**) and the voltage/the energy level of the capacitor (**b**), for: (1) 100 V; (2) 145 V; (3) 210 V—voltages applied to the test equipment.

The signals from the sensor output and the charging voltage of the capacitor are shown in Figure 16, where the increase in the amplitude of the signals and the energy of the capacitor according to the value of the applied alternating voltage are presented. The charging of the capacitor starts after the vibrations reach a minimum level, i.e., after a voltage of min. 145 V (case 2) is applied. That is why, in case 1, at 100 V, no charge is noticed. When the voltage exceeds 145 V (case 3), the amplitude of charge on the capacitor increases.

**Figure 17:**
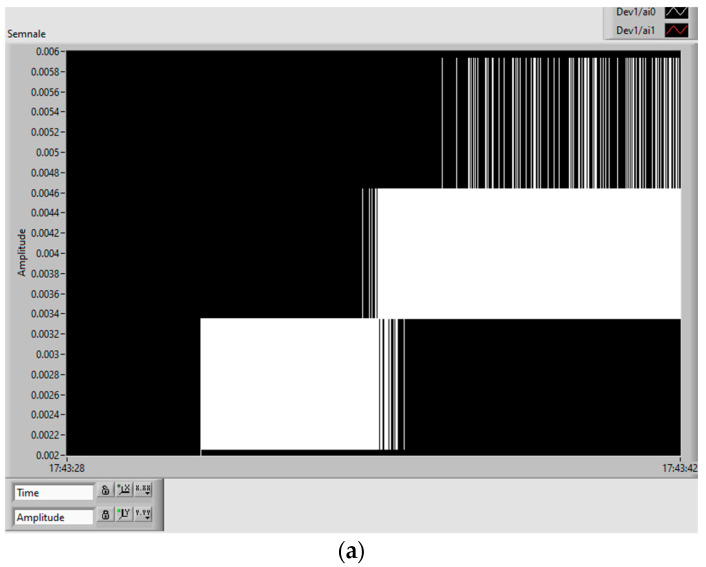

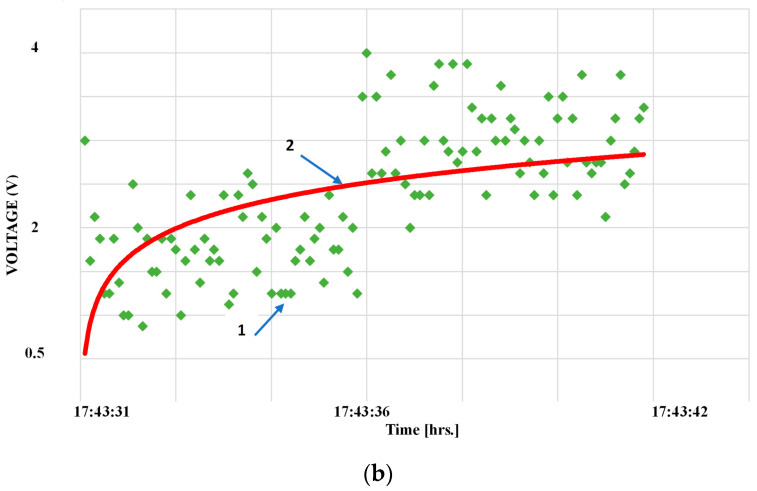
Digital signal on the capacitor (**a**) and charging characteristic (**b**), for the sensor without activation.

In Figure 17, this process is more explicitly explained, where in Figure 17b, 1–represents the individual charging voltage levels, and 2–represents the average charging characteristic of the capacitor. When associating the digital signal on the capacitor with the charging characteristic. Here, 1 represents the instant charging values from the sensor (green), and 2 represents the cumulative charging of the capacitor (red curve).

As seen in Figure 17, a minimum charging voltage is needed for the process to start, associated with the amplitude of the vibrations corresponding to 145 V, as explained above. The transient regime depends on the signal applied to the capacitor, and so the capacitor charges exponentially until it is theoretically close to full charge, then it will charge more and more slowly until it is fully charged. The charging characteristic shows the relatively long period of transient regime of capacitor charge, in this case 11 s for the sensor without electrical activation.

The following experiments are made with the sensor after the activation by the electric field. The sensor was activated by applying to its surfaces a continuous voltage of 31.6 V for 72 h to ensure saturation of the hysteresis effect.

Figure 16 shows the evolution of the signal on the capacitor when the vibrations increase.

**Figure 18:**
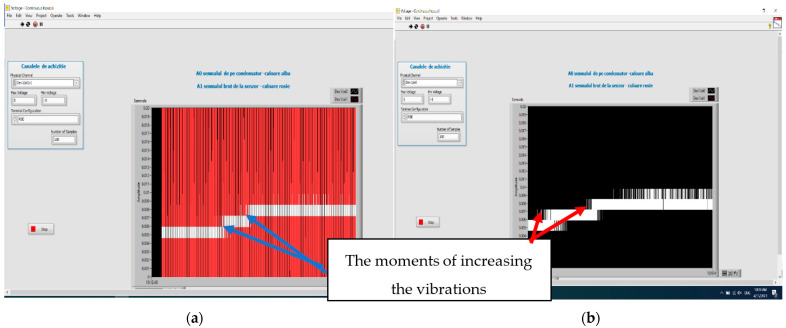
Vibration variation of the test equipment (**a**) and its influence upon the voltage on the capacitor (**b**), when the vibration increases on the activated sensor.

**Figure 19:**
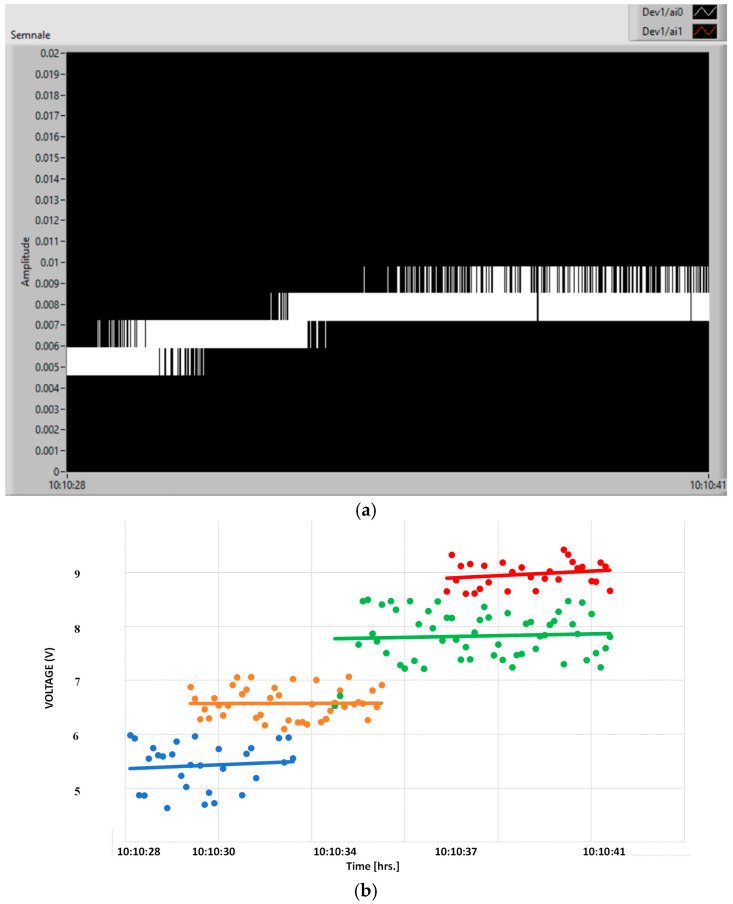
Digital signal on the capacitor (**a**) and charging characteristic (**b**) for the activated sensor.

Here also, the charging of the capacitor starts after the vibrations reach a minimum level, but much earlier compared to a non-activated sensor, i.e., after a voltage of min. 85 V is applied (Figure 18). In this case, even the transient regime is minimal, and the response to the increase in voltage in steps is immediate, as shown by the charging characteristic in Figure 19, where the voltage on the capacitor immediately increases to 5.5 V. In this specified case of excitation in 4 steps, the capacitor reaches a state close to full charge after about 13 s, e.g., 9 V.

At this point in the studies, the feature of ‘energy harvesting’ mode was demonstrated. By preliminary activating the sensor in the electric field, the value of the electric signal generated by the sensor increases, the efficiency of the sensor is higher, and the charging of the capacitor is faster.

An interesting aspect occurs when comparing the non-activated and activated sensors when the vibrations decrease.

**Figure 20:**
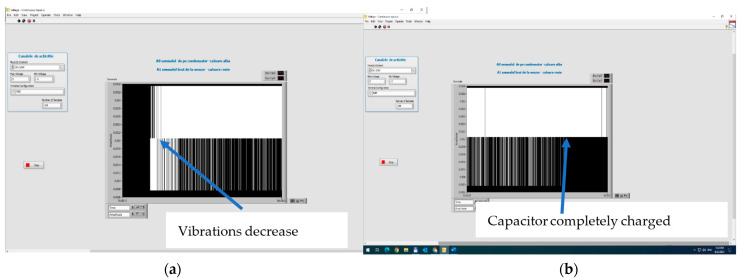
The charging of the capacitor when vibrations decrease: (**a**) capacitor on charge; (**b**) capacitor fully charged.

**Figure 21:**
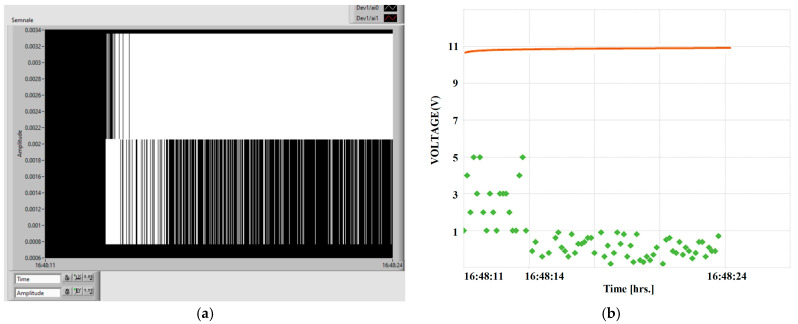
Digital signal on capacitor (**a**) and charging characteristic (**b**), when vibrations decrease for the activated sensor.

In the case of a non-activated sensor, when the applied voltage goes below 140 V, the sensor becomes passive, and the capacitor is not charged any more. However, in the case of an activated sensor, the charging of the capacitor continues even if the voltage is decreased to 70 V, until saturation (Figure 20) (when the black area becomes compact). In such cases, we can also speak about a mechanical hysteresis in the behavior of electrically activated matrix sensors. The conclusion is also sustained by analyzing the charging characteristic when vibrations decrease (Figure 21), where the charging of the capacitor continues until saturation. Here we practically reached the saturation charge of the capacitor, e.g., 11 V, even under the conditions when the vibrations decreased up to the limit of 1 V on the sensor.

## 6. Conclusions

The overall picture based on the actual literature shows that many researchers are carrying out research with the aim of testing piezoelectricity as the main source for energy harvesting, and the applications are very innovative, especially in the case of flexible piezoelectric systems.

Composite materials from polymer powders (TPU and TPO—herein polypropylene) with the addition of BaTiO_3_ until 35% were manufactured and interdisciplinary tested, and it was proved that the filler is well homogenized within the polymer structure with a uniform dispersion. For such composite material, in addition to glass transition processes and chemical oxidation processes, there is also a first-order phase transformation process—melting, due to the TPO (PP) melting process.

The addition of BaTiO_3_ dramatically increases the relative dielectric permittivity, from about 3 for pure polymers to about 10 for an addition of 12% BaTiO_3,_ and finally to about 30 for an addition of 35% BaTiO_3_. In regard to the dielectric loss, at lower frequencies the effect of interfacial polarization is detrimental, with a very significant increase for an addition of 35% BaTiO_3_. The piezoelectric characteristics of the composite with 35% BaTiO_3_, without preliminary activation under a tailored electric field, are remarkable, especially at lower frequencies.

The filament extruded from the composite material with 35% BaTiO_3_ was very elastic, close to the elasticity of rubber, but it provided good features to be used for 3D printing applications and presented a variation in a maximum diameter of 10%.

The importance and degree of novelty of our study consist in the demonstration of the operation in energy harvesting mode of the new concept of sensors made by 3D printing technology, made in the form of thermoplastic composites deposited in various forms; here, the testing was performed on a structure of rectangular network type, acting as a piezoelectric sensor in a dedicated signal processing circuit.

It was technically demonstrated that the 3D thermal deposition of composite filament with 35% BaTiO_3_ was a convenient process with no clogging of the nozzle, no problems with multilayer deposition, and no 3D printing defects.

For the development and integration of the signal processing circuits and the demonstration of the functionality of the energy harvesting features of 3D printed devices, an innovative test stand was developed and is assisted by a virtual instrument to produce and analyze the tailored vibrations to activate devices with functionality as piezoelectric sensors. As brief conclusions upon the electronic tests performed with non-activated and electrically activated devices:

- The electrically activated device has superior performance in terms of the generated electrical signal; the device with 35% BaTiO_3_ has an immediate and proportional response to periodic mechanical excitation.
- The device, with a higher concentration of BaTiO_3_ and a quasi-4D effect with frequency, responds better at lower frequencies to the variation of mechanical oscillations and achieves a quasi-uniform charging of the capacitor of the energy harvesting circuit, even if the mechanical oscillations become reduced in intensity.
- The higher the concentration of BaTiO_3_, the lower the minimum response threshold at low levels of mechanical oscillations, with an induced mechanical hysteresis effect when the mechanical oscillations decrease.

Such flexible piezoelectric systems can be used in various biomedical devices (as wearable electronics or intelligent prosthesis), generating enough energy to make such devices completely autonomous only by exploiting body movements at variable low frequencies.

## Figures and Tables

**Table 1:** I_HH_, I_HV_, I_VH_, and I_VV_ values of the PL spectra of the TPO:TPU 2:1 compound and its composites with BaTiO_3_ nanoparticles.

Compound	I_HH_ (counts/s)	I_HV_ (counts/s)	I_VH_ (counts/s)	I_VV_ (counts/s)
TPO:TPU 2:1	1.72 × 10^6^	1.8 × 10^6^	1.78 × 10^6^	2.17 × 10^6^
TPO:TPU 2:1 + 12% BaTiO_3_	1.22 × 10^5^	1.17 × 10^5^	1.13 × 10^5^	1.39 × 10^5^
TPO:TPU 2:1 + 25% BaTiO_3_	5.96 × 10^5^	6.08 × 10^5^	5.38 × 10^5^	6.87 × 10^5^
TPO:TPU 2:1 + 35% BaTiO_3_	6.99 × 10^4^	8.22 × 10^4^	7.73 × 10^4^	9.91 × 10^4^

**Table 2:** Setting the parameters of the laboratory 3D thermal printer.

Parameter	Grid
Layer height	0.2 mm
Angle of deposition	90
Deposit density	100%
Print speed	15 mm/s
Nozzle diameter	0.4 mm
Base temperature	60 °C
Extrusion temperature	190 °C
Turns	2
MULTIPLIER	1.2

## Data Availability

The data presented in this study are available on request from the corresponding author.
